# Symptoms of illness during travel and risk factors for non-adherence to malaria prophylaxis—a cross-sectional study in travellers from Germany

**DOI:** 10.1093/jtm/taad055

**Published:** 2023-04-25

**Authors:** Friederike Reinsberg, Mary W Moehlmann, Ralf Krumkamp, Lena Landsmann, Christian Heitkamp, Johannes Jochum, Marylyn Addo, Michael Ramharter, Christiane Radt, Camilla Rothe, Christof Vinnemeier, Benno Kreuels

**Affiliations:** Division of Infectious Diseases, First Department of Medicine, University Medical Center Hamburg-Eppendorf, Hamburg, Germany; Division of Infectious Diseases, First Department of Medicine, University Medical Center Hamburg-Eppendorf, Hamburg, Germany; Department of Infectious Disease Epidemiology, Bernhard-Nocht-Institute for Tropical Medicine, Hamburg, Germany; German Center for Infection Research (DZIF), Partner Site Hamburg—Lübeck—Borstel—Riems, Hamburg, Germany; Division of Infectious Diseases, First Department of Medicine, University Medical Center Hamburg-Eppendorf, Hamburg, Germany; Division of Infectious Diseases, First Department of Medicine, University Medical Center Hamburg-Eppendorf, Hamburg, Germany; Department of Tropical Medicine, Bernhard Nocht Institute for Tropical Medicine & I. Department of Medicine, University Medical Center Hamburg-Eppendorf, Hamburg, Germany; Division of Infectious Diseases, First Department of Medicine, University Medical Center Hamburg-Eppendorf, Hamburg, Germany; Department of Tropical Medicine, Bernhard Nocht Institute for Tropical Medicine & I. Department of Medicine, University Medical Center Hamburg-Eppendorf, Hamburg, Germany; Division of Infectious Diseases, First Department of Medicine, University Medical Center Hamburg-Eppendorf, Hamburg, Germany; Division of Infectious Diseases and Tropical Medicine, 4th Medical Department, LMU University Medical Centre, Munich, Germany; Department of Tropical Medicine, Bernhard Nocht Institute for Tropical Medicine & I. Department of Medicine, University Medical Center Hamburg-Eppendorf, Hamburg, Germany; Division of Tropical Medicine, First Department of Medicine, University Medical Center Hamburg-Eppendorf, Hamburg, Germany; Research Group Snakebite Envenoming, Division of Implementation Research, Bernhard-Nocht-Institute for Tropical Medicine, Hamburg, Germany

## Abstract

**Background:**

Perceived adverse effects of antimalarial chemoprophylaxis can be difficult to distinguish from travel-related illness and are often cited as important reasons for non-adherence or refusal of antimalarial chemoprophylaxis. We aimed to investigate the occurrence of symptoms of illness in travellers with and without chemoprophylaxis in a cross-sectional study after travel and to identify risk factors for non-adherence to prophylaxis.

**Methods:**

We enrolled 458 travellers to Africa and South America during their pre-travel medical consultation at the travel clinic of the University Medical Centre Hamburg-Eppendorf and conducted post-travel interviews on symptoms of illness and intake of malaria prophylaxis.

**Results:**

Eleven percent (49/437) of the participants reported symptoms of illness during travel. In total, 36% (160/448) of the participants reported prescription of chemoprophylaxis, the vast majority of these travelled to Africa (98%) and received atovaquone/proguanil (93%). Frequency of symptoms did not differ significantly between participants without prophylaxis and those taking atovaquone/proguanil. Non-adherence to prophylaxis was frequent (20%), but only 3% (4/149) of the participants stopped the medication early because of perceived side effects. Risk factors associated with non-adherence to prophylaxis included age under 30 years, travel to West or Central Africa and travel duration greater than 14 days.

**Conclusions:**

Symptoms of illness during travel occurred at similar frequencies irrespective of intake of chemoprophylaxis. Travellers should be informed about chemoprophylaxis in a balanced way, without raising fear of side effects, especially among groups at higher risk for incorrect use of prophylaxis.

## Background

Prior to the COVID-19 pandemic, travel to (sub) tropical destinations was a steadily growing market: in 2019, 43.6 million passengers travelled between Germany and intercontinental destinations, of which 5.9 million were German travellers to overseas destinations, including 0.83 million passengers to/from Africa and 0.65 million to/from Latin America.^1^^,^^2^ With the removal of travel restrictions, numbers of travellers are currently quickly increasing once more.^3^ In 2019, the Robert Koch Institute (RKI) reported 993 malaria cases in Germany with two deaths; 97% of those infected, contracted the disease in Africa.^4^ To reduce the number of malaria cases among travellers, the German Society for Tropical Medicine, Travel Medicine and Global Health (DTG) recommends antimalarial chemoprophylaxis with either atovaquone/proguanil, doxycycline or mefloquine for travellers to areas with a high risk of malaria transmission,^5^ in-line with most other international recommendations.^6–8^

Of the 993 malaria patients in Germany in 2019, 570 patients provided information on malaria prophylaxis: only 15% took chemoprophylaxis, and of these, only 32% reported continuous use.^4^ Even in travellers who take chemoprophylaxis, adequate adherence varies between 15 and 89%, depending on the population studied.^9–14^ Known risk factors for non-adherence or unwillingness to take prophylaxis include travel to rural areas, long-term travel, visiting friends and relatives (VFR), business travel, young age, a perceived low risk of malaria and, most importantly, a perceived high risk of adverse effects.^15^ In addition, adherence to chemoprophylaxis depends on the quality of the pre-travel consultation.^16^

All chemoprophylaxis regimens (atovaquone/proguanil, doxycycline and mefloquine) provide highly effective protection against malaria,^17^ but the frequency and spectrum of adverse effects varies.^18–20^ In a randomized trial with a placebo run-in phase, the lowest number of adverse effects was reported for atovaquone/proguanil and the highest for mefloquine. Abdominal pain, vomiting, indigestion and headache are the most common adverse effects of atovaquone/proguanil.^21^ The use of doxycycline is known to be associated with phototoxicity, indigestion and oesophageal ulcers.^22^^,^^23^ Mefloquine is contraindicated in case of previous mental illness due to the risk of neuropsychiatric side effects such as anxiety, depression and insomnia.^4^^,^^24^

Most of these adverse effects of chemoprophylaxis can be difficult to distinguish from travel-related illness, as 43–79% of all travellers who frequently travel to lower-middle-income countries develop symptoms of illness such as diarrhoea, respiratory infections and skin problems during travel.^25^ Therefore, symptoms of illness may be perceived as an adverse effect of chemoprophylaxis, although they might have also occurred without chemoprophylaxis, even if the duration and intensity of symptoms may differ. For ethical reasons, randomized controlled trials of chemoprophylaxis among travellers from non-endemic countries usually do not include a control group without prophylaxis. This study aimed to assess the frequency of symptoms of illness in travellers with and without antimalarial chemoprophylaxis and to identify potential risk factors for non-adherence in travellers attending the largest travel clinic in Northern Germany.

## Methods

The study was designed as a questionnaire-based cross-sectional study to assess the symptoms during travel and the association with malaria prophylaxis. As we also aimed to assess experience during border crossings in countries endemic for yellow fever (unpublished data), recruitment was restricted to travellers to Africa and South America. Areas where malaria prophylaxis is recommended and those with endemic yellow fever (YF) transmission largely overlap.

### Study site and study population

Participants were recruited at the travel clinic of the University Medical Centre Hamburg-Eppendorf at the Bernhard Nocht Institute for Tropical Medicine over four months from June 2017 onwards and interviewed by telephone until December 2018. Travellers over the age of 18 who planned to travel to or transit through certain African (Angola, Benin, Botswana, Burkina Faso, Burundi, Cameroon, Chad, Central African Republic, Congo, Côte d’Ivoire, Democratic Republic of Congo, Equatorial Guinea, Ethiopia, Gambia, Ghana, Guinea, Guinea Bissau, Kenya, Liberia, Mali, Mauritania, Niger, Nigeria, Rwanda, Senegal, Sierra Leone, Somalia, South Africa, South Sudan, Sudan, Tanzania, Togo, Uganda, Zambia and Zimbabwe) and South American countries (Argentina, Bolivia, Brazil, Colombia, Ecuador, French Guiana, Guyana, Panama, Paraguay, Peru, Suriname, Trinidad and Tobago and Venezuela) for a maximum of nine months were eligible for recruitment. Refusal of participation was the only exclusion criterion.

### Sample size

Due to the exploratory approach using a cohort of travellers a formal sample size calculation was not performed. The sample size was determined by the number of individuals who could be recruited within four months. With approximately 650 travellers to the above-mentioned countries attending our travel clinic per month and an expected participation rate of 30%, we anticipated recruitment of 800 participants over a four-month period, with an expected loss-to-follow-up of 20%.

A post hoc calculation was conducted to estimate the statistical power for a two-proportion test with different sample sizes assuming a two-sided test with an alpha-level of 5%. In the primary analysis, the frequency of symptoms in individuals with (*n* = 149) and without (*n* = 288) chemoprophylaxis was compared. Based on a symptom frequency of 10% in travellers without and a probability of symptoms of 15, 20 or 30% in travellers with prophylaxis, the statistical power of a test would be 32, 80 and >99%, respectively.

### Data collection

Individuals eligible for participation were informed about the study by their attending physician during the pre-travel consultation. Consenting participants provided their name, contact details, destination country and travel dates on a pre-travel data collection form marked with an alphanumeric code. As soon as possible after return from travel, participants were contacted for a telephone interview by the study team. A maximum of five attempts to contact participants was undertaken. The interview was based on a standardized, paper-based questionnaire (Supplementary Figure 1) marked with the above-mentioned alphanumeric code. Data collected included age, sex, nationality, travel dates, destination, use of malaria chemoprophylaxis (medication, adherence, perceived side effects) and the occurrence of the following symptoms: diarrhoea, abdominal pain, vomiting, nausea, sunburn/photosensitivity, nightmares, vertigo, visual disorder, low mood or fatigue. The paper-based questionnaires were transferred into an electronic version using Epidata (version 4.4.2.1).

### Data analysis

We examined the relationship between the prescription of chemoprophylaxis, chemoprophylaxis regimen and traveller- or travel-related factors. Discrete variables were expressed as absolute and relative frequencies, continuous variables as median with interquartile range (IQR). African regions were classified according to the United Nations geoscheme for Africa.^26^ We followed the DTG’s assessment of the malaria transmission risk for each country.^5^ Correct intake of chemoprophylaxis was classified according to the reported start and end date as well as the frequency of medication intake following the DTG guidelines.^5^ Fisher’s exact test was used to compare the frequency of disease symptoms between travellers without prophylaxis versus travellers who took atovaquone/proguanil. To identify risk factors for non-adherence, we estimated the odds ratio (OR) and corresponding 95% confidence interval (CI) using bivariable logistic regression models. Variables considered for the bivariable analysis were included in a multivariable logistic regression model to control for potential confounding. No further model selection procedures were applied. Regression analysis was limited to travellers who took chemoprophylaxis and provided information on prophylaxis intake. In two independent variables (i.e. travel duration and perception of side effects) missing values were observed, which were imputed for the regression analysis using a random forest model. Data analysis was performed using R (version 4.1.3) and the missForest package (version 1.5) was used to impute missing values.

### Ethical approval

The study was approved by the Ethics Committee of the Hamburg Board of Physicians (approval number: PV5520–3828-BO-ff).

## Results

### Study population

**Figure 1:**
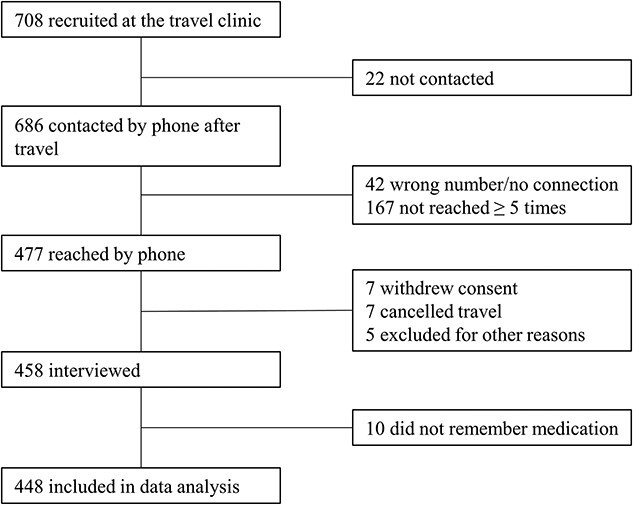
Flowchart of recruitment of study participants Depiction of number of enrolled participants, number of participants included in data analysis and reason for exclusion

A total of 708 participants were recruited to the study before travel. The recruitment and selection process are illustrated in Figure 1. In total, we were able to reach 477 (67%) participants by phone after travel. Of these, 19 were excluded because they withdrew their consent, cancelled their travel, changed the travel date or were not able to participate. We conducted 458 telephone interviews in German or English language. After excluding participants who did not remember their chemoprophylaxis regimen, 448 participants were included in the data analysis.

Characteristics of the study participants are summarized in Table 1. About, 220 (49%) of the study participants were female. The median age was 37 (IQR: 29–52) years and the vast majority (*n* = 427; 95%) were German nationals. About, 213 (48%) participants travelled to South America and 235 (52%) to Africa. Of the latter, 67 (29%) travelled to West or Central Africa, 87 (19%) participants travelled to countries with permanently high risk of malaria transmission and 330 (74%) to countries with seasonal or local high risk of transmission. The median duration of travel was 19 (IQR: 14–30) days. Data on the date and duration of the trip were missing in 12 participants.

**Table 1:** Characteristics of cohort

	All (*N* = 448)	No chemo-prophylaxis (*N* = 288)	Atovaquone/Proguanil (*N* = 149)	Doxycycline (*N* = 5)	Mefloquine (*N* = 6)
Sex Female Male	220 228	146 142	71 (96%) 78 (91%)	1 (1%) 4 (5%)	2 (3%) 4 (5%)
Age [median (IQR)] < 30 ≥30	37 (29, 52) 117 331	36 (28, 51) 78 210	40 (29, 52) 39 (100%) 110 (91%)	48 (44, 53) 0 (0%) 5 (4%)	37 (33, 48) 0 (0%) 6 (5%)
Nationality German Non-German	427 21	274 14	143 (93%) 6 (86%)	5 (3%) 0 (0%)	5 (3%) 1 (14%)
Destination South America Africa	213 235	210 78	3 (100%) 146 (93%)	0 (0%) 5 (3%)	0 (0%) 6 (4%)
Region in Africa West/Central Africa Other	67 168	8 70	53 (90%) 93 (95%)	2 (3%) 3 (3%)	4 (7%) 2 (2%)
Malaria transmission in destination country High risk Seasonally or locally high risk Low or no risk	87 330 31	11 246 31	67 (88%) 82 (98%) 0 (0%)	3 (4%) 2 (2%) 0 (0%)	6 (8%) 0 (0%) 0 (0%)
Duration [median (IQR)] ≤ 14 > 14 missing	19 (14–30) 136 300 12	21 (15–33.5) 69 210 9	15 (12–21) 64 (96%) 82 (91%) 3 (100%)	21 (19–25) 1 (1%) 4 (4%) 0 (0%)	27 (16.5–33) 2 (3%) 4 (4%) 0 (0%)

Based on comprehensive medical advice and traveller choice, 160 (36%) participants reported prescription of antimalarial chemoprophylaxis: 149 (93%) received atovaquone/proguanil, five (3%) doxycycline and six (4%) mefloquine (Table 1). Only participants older than 30 years were given doxycycline or mefloquine. Only three travellers to South America reported receiving a prescription for chemoprophylaxis, in contrast to 157 (67%) travellers to Africa. More travellers to West and Central Africa (59/67; 88%) than to other African regions (98/168; 58%) received chemoprophylaxis. Among travellers to countries with high risk of malaria transmission, 76 (87%) reported prescription of chemoprophylaxis. We were able to track the travel advice documents for 8 of the 11 participants who indicated that they were not advised to take chemoprophylaxis despite travel to a high risk area. In seven cases, it was documented that chemoprophylaxis had been recommended or prescribed. One participant only spent one night in Senegal before travelling to Cape Verde and chemoprophylaxis was not recommended in this case.

### Occurrence of potential side effects

**Figure 2:**
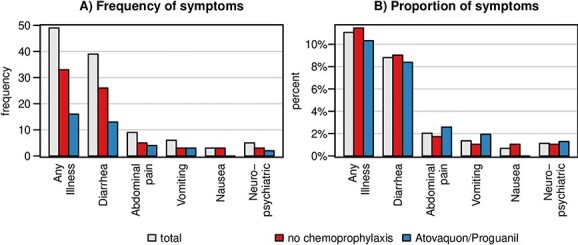
Frequency of symptoms by medication absolute (A) and relative (B) frequencies of symptoms among participants without chemoprophylaxis (*n* = 288) and participants receiving atovaquone/proguanil (*n* = 149)

To determine the occurrence of side effects, we compared self-reported symptoms of illness during travel between participants with and without chemoprophylaxis (Figure 2, Supplementary Table 1). We focused the analysis on travellers without chemoprophylaxis (*n* = 288) compared to those taking atovaquone/proguanil (*n* = 149), as few travellers received mefloquine or doxycycline (data for all drugs shown in Supplementary Table 2). Among the 437 participants analysed, 11% (*n* = 49) reported a symptom of illness during travel. Diarrhoea (*n* = 39; 9%) was the most common symptom, followed by abdominal pain (*n* = 9; 2%) and vomiting (*n* = 6; 1%). All other symptoms (nausea and neuropsychiatric symptoms such as vertigo and low mood/fatigue) were present in less than 1% of the travellers. Symptoms that were not reported by any participant included sunburn/higher photosensitivity, nightmares and visual disturbances. There was no significant difference in the frequency of symptoms in travellers not taking prophylaxis and those taking atovaquone/proguanil (Supplementary Table 1).

### Potential confounding factors

Due to the small number of participants per group for each symptom category, it was not possible to apply multivariable logistic regression models to adjust for potential confounders Instead, we assessed the association of potential confounders with the exposure (atovaquone/proguanil) and with the outcome (symptoms of illness) through cross-tabulation. Relevant associations were seen for travel destination and duration of travel (Table 1).

While 98% (*n* = 146) of participants who received atovaquone/proguanil travelled to Africa, only 27% (*n* = 78) of participants without chemoprophylaxis reported Africa as their travel destination. We did not find any difference regarding the frequency of symptoms in participants travelling to Africa vs South America, which ruled out travel destination as a confounding factor (Supplementary Table 3). There was no difference in frequency of symptoms in short-term (≤14 days) and long-term travellers (>14 days) (Supplementary Table 4). Therefore, a potential confounding by these variables seems unlikely.

### Adherence to malaria prophylaxis

About, 158 (99%) of the participants who reported prescription of chemoprophylaxis provided information on adherence. Among these 126 (80%) reported to have taken their medication correctly. Incorrect intake included: 5% (*n* = 8) not taking the medication at all, 3% missing single doses (*n* = 5), 9% (*n* = 15) stopping the medication too early and other intake errors (*n* = 4; 3%). Overall, of 149 participants taking atovaquone/proguanil, seven (5%) did not take the prophylaxis at all, four (3%) discontinued prophylaxis due to perceived side effects, six (4%) stopped the medication early for other reasons, five (3%) forgot single doses and three (2%) reported other intake errors. Of the five participants taking doxycycline, four reported incorrect intake: one did not take the prophylaxis at all, one discontinued the medication due to perceived side effects, one stopped the medication early for other reasons and one reported another intake error. Of the six patients taking mefloquine, one discontinued the medication due to perceived side effects and two stopped the medication early for other reasons. Perception of side effects occurred both in travellers who took the prophylaxis correctly (17/126; 13%) and in those who did not (6/32; 19%). The symptoms perceived as side effects mainly included gastrointestinal complaints (*n* = 10) as well as sleep disturbances (*n* = 7) and generally feeling unwell (*n* = 5). None of the participants reported being diagnosed with or treated for malaria.

To identify risk factors for incorrect intake, we conducted a bivariable and multivariable logistic regression analysis (Table 2). In bivariable analysis, we found some evidence of an association between incorrect intake of prophylaxis and age under 30 years (OR = 2.3; 95%-CI: 1.0–5.3), travel to West or Central Africa (OR = 2.3; 95%-CI: 1.0–5.0) and travel duration > 14 days (OR = 3.1; 95%-CI: 1.3–8.4). In the multivariable regression, accounting for the other predictors, the adjusted OR for perception of side effects rose to 2.2 (95%-CI: 0.7–6.3); however, the wide CI indicate the low precision of the calculation. The other estimates did not change substantially in the multivariable analysis.

**Table 2:** Factors associated with non-adherence to prescribed chemoprophylaxis (*N* = 158)

	Incorrect intake (*N* = 32)	Correct intake (*N* = 126)	Crude model [OR (95%-CI)]	Multivariable model [aOR (95%-CI)]
Sex Female Male	12 (16%) 20 (24%)	62 (84%) 64 (76%)	0.6 (0.3–1.4) Ref.	0.6 (0.2–1.3) Ref.
Age <30 ≥30	12 (32%) 20 (17%)	26 (68%) 100 (83%)	2.3 (1.0–5.3) Ref.	2.5 (1.0–6.3) Ref.
Destination West/Central Africa Other destination	17 (29%) 15 (15%)	42 (71%) 84 (85%)	2.3 (1.0–5.0) Ref.	2.8 (1.2–6.8) Ref.
Travel duration^a^ ≤ 14 >14	7 (11%) 25 (27%)	59 (89%) 67 (73%)	Ref. 3.1 (1.3–8.4)	Ref. 3.3 (1.3–9.3)
Perception of any side effect^b^ Yes No	7 (28%) 25 (19%)	18 (72%) 108 (81%)	1.7 (0.6–4.5) Ref.	2.2 (0.7–6.3) Ref.

^a^Three missing values imputed

^b^Five missing values imputed

## Discussion

Our study on malaria chemoprophylaxis in travellers from a non-endemic country revealed three key findings: First, the frequency of symptoms during travel did not differ markedly between travellers with and without prophylaxis. Second, perceived side effects were only reported by 3% of travellers taking atovaquone/proguanil as reason for discontinuation of chemoprophylaxis. Third, we found evidence that factors such as young age, long-term travel and travel to West/Central Africa were associated with non-adherence to prophylaxis, which could support targeted pre-travel advice.

Eleven percent of participants reported becoming ill while travelling. A meta-analysis of studies looking at the prevalence of symptoms among travellers to mainly low-middle income countries found a range of 43–79% of travellers falling ill during or after travel in the four studies providing the best estimate, while the prevalence was 6–87% across all studies.^25^ This wide range can be explained by differences in the focus on specific symptoms, as well as by different survey methods. Our estimate is below this range, possibly due to the destinations included and the way we assessed symptoms. Unlike most other studies, we only focused on symptoms that occurred during the trip. Furthermore, the retrospective assessment of symptoms during travel through a telephone interview after return may have led to mild or transient symptoms not being reported. Analyses based on post-travel telephone interviews showed a prevalence of symptoms of 39–64%,^27–29^ while studies in which a symptom diary was kept found a prevalence of 64–79%,^30^^,^^31^ suggesting that travellers do not remember mild symptoms if the travel was not substantially impaired. Questions about symptoms of illness and chemoprophylaxis were also asked only in the second half of the interview. Therefore, participants may have been less detailed or precise in their responses. In addition, several studies have shown that the prevalence of symptoms was markedly increased when travelling to the Indian subcontinent compared to other destinations.^27^^,^^30^^,^^32^ Unlike most other studies on travel-related illnesses, the current study focused on travellers to YF-endemic countries, thus excluding travellers to Asia, which may have led to a selection bias. Diarrhoea, followed by other gastrointestinal symptoms, was the most common complaint. This is consistent with most studies in the literature, which finds that gastrointestinal symptoms are the most common symptoms among travellers, followed by respiratory symptoms.^25^^,^^32^^,^^33^

Interestingly, in the current study, the prevalence of symptoms did not differ markedly between travellers taking chemoprophylaxis, i.e. atovaquone/proguanil, and those without prophylaxis. In two studies of long-term use (more than four weeks) of atovaquone/proguanil for malaria prophylaxis without a control group, the frequency of diarrhoea, the most common symptom, was 18–29%,^21^^,^^34^ which is higher than in this cohort. As the frequency of diarrhoea did not differ significantly from travellers with other prophylaxis regimens (mefloquine, chloroquine/proguanil), the occurrence of diarrhoea was interpreted as a travel-associated illness in one of the studies.^34^ However, in the same study, two out of 154 participants discontinued prophylaxis with atovaquone/proguanil due to diarrhoea, which subsided after stopping the medication. Moreover, in a study of French travellers to Senegal, 37% of travellers with gastrointestinal symptoms attributed their symptoms to the antimalarial medication, and gastrointestinal disturbances were associated with poor adherence to prophylaxis.^35^ The predominant prescription of atovaquone/proguanil in our study reflects a survey of European travel medicine experts, 92% of whom would recommend atovaquone/proguanil for a traveller without comorbidities travelling for two weeks.^36^ Overall, symptoms of illness can occur during travel regardless of prophylaxis. Pre-travel advice should therefore provide a differentiated view of travel-related illnesses and the effects and side effects of malaria prophylaxis.

Only 87% of the study participants who travelled to countries with a high risk of malaria reported having received a prescription for antimalarial chemoprophylaxis at their pre-travel medical consultation, although chemoprophylaxis is generally recommended for these areas and was also recommended to these travellers. It remains unclear what may have led to this deviation from the recommendations. A subjectively perceived low risk of malaria or its severity by some travellers, may have led to an unwillingness to accept a prescription or led them to forget that they had been offered a prescription. Some participants may have visited our travel clinic primarily for YF vaccination and not for a general pre-travel medical advice and therefore may have forgotten that a malaria prophylaxis was recommended. This is in line with a study of family adherence to pre-travel advice in France that showed that 100% of children were vaccinated against YF, but only 93% purchased the recommended chemoprophylaxis.^37^ A survey of air travellers from Amsterdam to Ghana showed a similar discrepancy, with 67.5% seeking pre-travel medical advice but only 60% purchasing prophylaxis.^38^

About, 80% of travellers reported using chemoprophylaxis correctly, while 5% did not take the medication at all. This is comparable to a sample of international travellers at Harare/Victoria Falls Airport, where 23% did not carry chemoprophylaxis.^10^ Other studies combining recruitment at pre-travel consultation with a post-travel survey found similar adherence rates of 79–89%.^12^^,^^13^ Since our study was based on retrospective self-reporting, adherence may have been overestimated, as shown in a study comparing data from questionnaires (adherence rate 48%) and electronic pill boxes (32%).^11^ Adherence to chemoprophylaxis may also be higher among persons who participate in a study and telephone interview, remember the name of their medication and provide information on intake.

Incorrect intake of prophylaxis was associated with age under 30 years, travel to West/Central Africa and travel duration greater than 14 days, generally reflecting the results of a recent systematic review.^15^ Although younger age has been associated with non-adherence in several studies, the cut-off values ranged widely from 23 to 60 years, depending on the study.^15^^,^^38–44^ Since our study did not collect data on travel purpose or style, a higher proportion of travellers who were VFR among travellers to West Africa^45^ and among long-term travellers^46^ may have confounded this association. VFR have cited the cost of medical visits and chemoprophylaxis as a barrier to malaria prevention, although travellers on longer trips effectively save on health care expenses if they follow prophylaxis recommendations.^47^^,^^48^ Targeted pre-travel advice should therefore focus on the specific needs and challenges of groups at higher risk of incorrect prophylaxis use.

Our study has several limitations. We focussed only on travellers to YF-endemic countries and did not include all travellers attending the travel clinic. While it is possible that the results may therefore not be generalizable to all travellers, there is a large overlap between areas where chemoprophylaxis is recommended and YF-endemic areas and it seems unlikely that the frequency of side effects due to chemoprophylaxis is affected by travel destination. Compared to a similar cross-sectional study based on telephone interviews with a response rate of 84%,^12^ the response rate of 71% (458 interviews among 644 participants with correct telephone number) in this study was relatively low. This may have led to a bias towards more compliant travellers, or those with a higher interest in participation due to perceived side effects. However, we did not find a particularly high frequency of side effects and our results are comparable to previous, smaller studies, making a strong selection bias unlikely.^12^^,^^13^ Finally, as our study did not include data on participants’ socioeconomic status, travel purpose and VFR status, we were not able to investigate in detail, which factors contributed to adherence to chemoprophylaxis.

Our results provide data for improved communication in the pre-travel consultation: symptoms during travel were reported with similar frequency, irrespective of malaria prophylaxis and travellers should be informed about chemoprophylaxis in a balanced way without raising fear of side effects. A special counselling focus on adherence should be placed on young travellers, travellers to West/Central Africa and long-term travellers.

## Funding

This work was supported by institutional funding of the University Medical Centre Hamburg-Eppendorf (UKE).

## Authors’ contributions

M.W.M., C.Ro., C.V. and B.K. planned the study. M.W.M., B.K., C.Ro. and C.V. designed the questionnaire. M.W.M., L.L., C.Ra., C.H. and J.J. recruited the participants. F.R. and R.K. analyzed the data. F.R. and B.K. wrote the first draft of the paper. M.A. and M.R. reviewed the first draft of the paper and made important contributions to revising the manuscript. All authors contributed to the article and approved the final version.

## Conflict of interest

The authors have declared no conflict of interest.

## Data availability

The data underlying this article will be shared on reasonable request to the corresponding author.

## Grant support

None.

## Supplementary Material

Supplementary_Data_taad055
