# Supplementary material for: Symptoms of illness during travel and risk factors for non-adherence to malaria prophylaxis—a cross-sectional study in travellers from Germany
Source: J Travel Med. 2023 Apr 25;30(3):taad055. doi: 10.1093/jtm/taad055 (PMC10198432; doi:10.1093/jtm/taad055)
Supplement: Supplementary_Data_taad055 [file supplementary_data_taad055.docx]

**Symptoms of illness during travel and risk factors for non-adherence to malaria prophylaxis – a cross-sectional study in travellers to Africa and South America**

Friederike Reinsberg, Mary Wambui Moehlmann, Ralf Krumkamp, Lena Landsmann, Christian Heitkamp, Johannes Jochum, Marylyn Addo, Michael Ramharter, Christiane Radt, Camilla Rothe, Christof Vinnemeier, Benno Kreuels

**Supplementary Figure 1: Questionnaire**


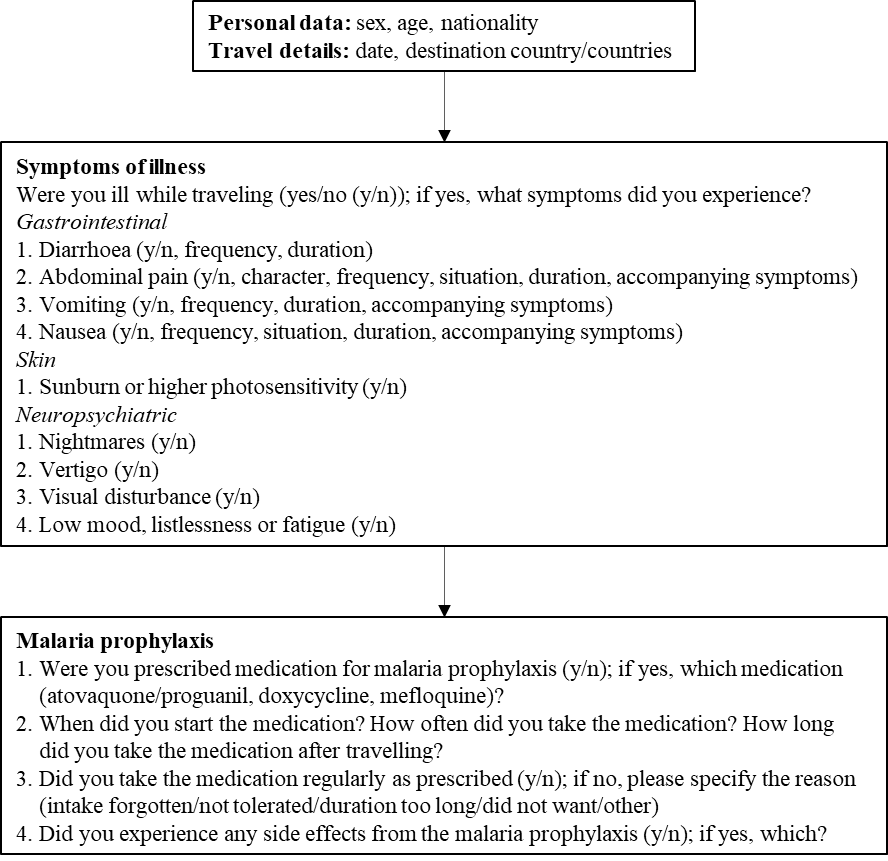


**Supplementary Table 1: Frequency of symptoms in travellers Atovaquone/Proguanil vs. no prophylaxis**

|  | Total  (N= 437) | No chemoprophylaxis  (N=288) | Atovaquone/Proguanil  (N=149) | p |
| --- | --- | --- | --- | --- |
| Any Illness | 49 (11%) | 33 (11%) | 16 (11%) | 0.88 |
| Diarrhoea | 39 (9%) | 26 (9%) | 13 (9%) | 1 |
| Abdominal pain | 9 (2%) | 5 (2%) | 4 (3%) | 0.50 |
| Vomiting | 6 (1%) | 3 (1%) | 3 (2%) | 0.42 |
| Nausea | 3 (1%) | 3 (1%) | 0 (0%) | 0.55 |
| Neuropsychiatric | 5 (1%) | 3 (1%) | 2 (1%) | 1 |

**Supplementary Table 2: Frequency of symptoms in travellers for all groups**

|  | No chemo-prophylaxis  (N=288) | Atovaquone/Proguanil  (N=149) | Doxycycline  (N=5) | Mefloquine  (N=6) |
| --- | --- | --- | --- | --- |
| Any Illness | 33 (11%) | 16 (11%) | 1 (20%) | 0 (0%) |
| Diarrhoea | 26 (9%) | 13 (9%) | 1 (20%) | 0 (0%) |
| Abdominal pain | 5 (2%) | 4 (3%) | 0 (0%) | 0 (0%) |
| Vomiting | 3 (1%) | 3 (2%) | 0 (0%) | 0 (0%) |
| Nausea | 3 (1%) | 0 (0%) | 0 (0%) | 0 (0%) |
| Neuropsychiatric | 3 (1%) | 2 (1%) | 0 (0%) | 0 (0%) |

**Supplementary Table 3: Frequency of symptoms in travellers by continent**

|  | Total  (N=448) | Africa (N=235) | South America (N=213) | p |
| --- | --- | --- | --- | --- |
| Any Illness | 50 (11%) | 25 (11%) | 25 (12%) | 0.77 |
| Diarrhoea | 40 (9%) | 20 (9%) | 20 (9%) | 0.87 |
| Abdominal pain | 9 (2%) | 4 (2%) | 5 (2%) | 0.74 |
| Vomiting | 6 (1%) | 5 (2%) | 1 (0%) | 0.22 |
| Nausea | 3 (1%) | 1 (0%) | 2 (1%) | 0.61 |
| Neuropsychiatric | 5 (1%) | 2 (1%) | 3 (1%) | 0.67 |

**Supplementary Table 4: Frequency of symptoms by travel duration**

|  | (N=436)^a^ | ≤14 days (N=136) | >14 days (N=300) | p |
| --- | --- | --- | --- | --- |
| Any Illness | 47 (11%) | 11 (8%) | 36 (12%) | 0. 32 |
| Diarrhoea | 39 (9%) | 8 (6%) | 31 (10%) | 0.20 |
| Abdominal pain | 8 (2%) | 1 (1%) | 7 (2%) | 0.44 |
| Vomiting | 5 (1%) | 1(1%) | 4 (1%) | 1.00 |
| Nausea | 2 (0%) | 2 (1%) | 0 (0%) | 0.10 |
| Neuropsychiatric | 4 (1%) | 1 (1%) | 3 (1%) | 1.00 |

^a^12 missing values
